# Tracking Ground Targets with a Road Constraint Using a GMPHD Filter

**DOI:** 10.3390/s18082723

**Published:** 2018-08-18

**Authors:** Jihong Zheng, Meiguo Gao

**Affiliations:** School of Information and Electronics, Beijing Institute of Technology, Beijing 100081, China; meiguo_g@bit.edu.cn

**Keywords:** Gaussian mixture probability hypothesis density filter, directional process noise, state constraint, ground moving target tracking, cardinalized PHD filter, labeled multi-Bernoulli filter

## Abstract

The Gaussian mixture probability hypothesis density (GMPHD) filter is applied to the problem of tracking ground moving targets in clutter due to its excellent multitarget tracking performance, such as avoiding measurement-to-track association, and its easy implementation. For the existing GMPHD-based ground target tracking algorithm (the GMPHD filter incorporating map information using a coordinate transforming method, CT-GMPHD), the predicted probability density of its target state is given in road coordinates, while its target state update needs to be performed in Cartesian ground coordinates. Although the algorithm can improve the filtering performance to a certain extent, the coordinate transformation process increases the complexity of the algorithm and reduces its computational efficiency. To address this issue, this paper proposes two non-coordinate transformation roadmap fusion algorithms: directional process noise fusion (DNP-GMPHD) and state constraint fusion (SC-GMPHD). The simulation results show that, compared with the existing algorithms, the two proposed roadmap fusion algorithms are more accurate and efficient for target estimation performance on straight and curved roads in a cluttered environment. The proposed methods are additionally applied using a cardinalized PHD (CPHD) filter and a labeled multi-Bernoulli (LMB) filter. It is found that the PHD filter performs less well than the CPHD and LMB filters, but that it is also computationally cheaper.

## 1. Introduction

Ground target tracking includes the track establishment and maintenance of ground moving targets, such as vehicles and convoys. Establishing and maintaining tracks of relevant ground moving targets is particularly challenging in dense target scenarios, mainly due to the sensor’s Doppler blind zone, terrain obscuration, high clutter, and other factors. These factors can cause a drastic deterioration in tracking performance and even result in track loss. Since ground target tracking is such a challenging issue, all available information resources must be exploited, including the sensor data themselves, as well as contextual knowledge about the underlying scenario. For ground target tracking, typical examples of contextual knowledge are digital roadmaps provided by geographic information system (GIS), which should be incorporated into the target tracking and sensor data fusion process.

In many ground target tracking applications, roadmap information is exploited to enhance the tracking performance in aspects such as track quality and track continuity of road moving targets [1]. The state-of-the-art sensor technology for such an application is airborne ground moving target indicator (GMTI) radar [2]. The ground target tracking algorithms for integrating roadmap information with GMTI radar measurements in the track estimation process are classified into two main categories. One of these categories is based on a data association mechanism and roadmap-assisted single target filtering approach. For example, in Ref. [3], roadmap information is integrated into a variable structure interacting multiple model (VS-IMM) extended Kalman approach, where filter modules are adaptively modified, added, or removed. Two-dimensional assignment is selected as the data association mechanism to decide which GMTI measurements are to be used to update each track. Similarly, in Ref. [4], the authors propose and evaluate a ground target tracking algorithm for integrating roadmap information with GMTI radar measurements, in a variable structure multiple model particle filter. In Ref. [5], the multiple hypotheses tracking (MHT) method is chosen to solve the data targets assignment problem for ground target tracking. Other applications of the data association mechanism can be found in Ref. [6,7,8,9].

The second main category of ground target tracking algorithm is based on the theory of the random finite set (RFS) [10,11,12], which has attracted substantial interest in recent years because of the development of the probability hypothesis density (PHD) filter [13,14], cardinalized PHD (CPHD) filter [15,16,17], and multi-target Bernoulli filter [18,19,20,21,22,23,24]. The PHD filter is an approximate method proposed to solve the computational difficulties in the multi-target Bayesian filter, and has attracted much attention from researchers due to the following advantages: (1) the potential to reduce computational complexity; (2) the fact that statistical models for observations such as missed detections, sensor field of view (FoV), and false alarms (often assumed to be a Poisson distribution) can be explicitly integrated; (3) it offers a statistical model that explicitly integrates multi-target dynamic mechanisms, including target disappearance and target appearance; (4) it can be implemented by using both Gaussian mixing and sequential Monte Carlo approximation techniques; (5) it avoids measurement-to-track association; and (6) due to its excellent clutter-rejection ability, the PHD filter can directly estimate the target number from the data at each step.

The PHD filter greatly alleviates the computational load of a completely multi-target Bayesian filter. Even so, it still involves multidimensional integration, that must be approximated using some implementation method. Generally, the PHD can be implemented by using both Gaussian mixing and sequential Monte Carlo approximation techniques, because in principle, the sequence Monte Carlo (SMC) implementation of the PHD filter [25,26,27,28] can adapt to any Markov target dynamic model. Nevertheless, the SMC method has some inherent flaws, that is, it requires many particles and there is unreliability in the clustering algorithm for state extraction [26,29]. However, these problems are mitigated in the Gaussian Mixture (GM) implementation of the PHD filter [29,30,31,32,33,34,35,36]. In Ref. [37], the GMPHD filter is applied to solve the problem of tracking ground moving targets, and the roadmap information is developed to enhance the track and track continuity. The main drawback of this approach is that the PHD prediction is performed in the road coordinate system, while the PHD update is performed in the Cartesian ground coordinate system. This coordinate transformation process increases the complexity of the algorithm and reduces computational efficiency.

In this paper, we present two non-coordinate transformation approaches based on the theory of the PHD filter, integrating roadmap information with GMTI radar measurements in the track filter process, and the target dynamics of the filters which are constrained to the road in the tracking scenario. The first approach uses the directional process noise (DPN) GMPHD filter, dubbed the DPN-GMPH filter, in which roadmap information is used to handle the motion along a road. In other words, for an on-road target, the road constraint means more uncertainty in the along-road direction than orthogonal to it. A description of the DNP method can be found in Ref. [38]. The second approach is the state constraint (SC) GMPHD filter, hereafter referred to as the SC-GMPHD filter, in which the roadmap information is used to first establish the constraint relationship among the position parameters in the target state, and then the constraint relationship is applied to modify the result of the target state estimation. Other descriptions of the SC method can be found in the literature [39,40,41,42,43,44]. The application of the SC method in these studies are all based on the traditional data association method, not the random finite set. Additionally, the GMTI radar measurements at each step are employed to update the intensity of the GMPHD filter. Furthermore, a road-constrained method combined with DPN and SC methods is presented, in which the DPN and SC methods are applied to the prediction step and update step of the same filter, respectively. The road constraint method is subsequently applied to the PHD, CPHD and labeled multi-Bernoulli (LMB) filters.

This paper is organized as follows: Section 2 presents the Gaussian mixture probability density (GMPHD) filter recursion; in Section 3, we describe three types of GMPHD filter incorporating map information, and demonstrate the capability of the proposed algorithm through simulations; in Section 4, the proposed methods are also used with the CPHD and LMB filters, and a curved road tracking scenario is used to evaluate the performance of the three kinds of filters; and Section 5 summarizes the main conclusions of this work.

## 2. GMPHD Filter

For a linear Gaussian multi-target model, the PHD recursion [29] admits a closed-form solution. The linear Gaussian multi-target model is summarized below:

(1) The single-target Markov transition density and likelihood function are both linear Gaussian, that is:
  (1) $$f_{k|k-1}\left(x|x^{\prime }\right)=N\left(x;F_{k-1}x^{\prime },Q_{k-1}\right)$$
  (2) $$g_{k}\left(z|x\right)=N\left(z;H_{k}x,R_{k}\right)\;$$
  where N(⋅;m,P) denotes a Gaussian probability density function with mean m and covariance P; $F_{k-1}$ and $H_{k}$ are the state transition and measurement matrices, respectively; $Q_{k}$ and $R_{k}$ denote the corresponding covariance matrices of process noise and measurement noise respectively; x and $x^{\prime }$ are the target state vectors of the current time and the previous time, respectively; z is the measurement vector, and k is the time step.
(2) The probabilities of target survival and detection are both independent of the kinematic state, i.e.:
  (3) $$p_{S,k}\left(x\right)=p_{S,k}\;$$
  (4) $$p_{D,k}\left(x\right)=p_{S,k}$$
(3) The intensity of birth RFS is a Gaussian form:
  (5) $$\gamma_{k}\left(x\right)=\sum_{i=1}^{J_{\gamma ,k}}w_{\gamma ,k}^{\left(i\right)}N\left(x;m_{\gamma ,k}^{\left(i\right)},P_{\gamma ,k}^{\left(i\right)}\right)$$
  where $J_{\gamma ,k},w_{\gamma ,k}^{\left(i\right)},m_{\gamma ,k}^{\left(i\right)},P_{\gamma ,k}^{\left(i\right)},i=1,\cdots ,J_{\gamma ,k}$ are given model parameters of the intensity of birth.

For the linear Gaussian multi-target model, the following two propositions show how the Gaussian components of the posterior intensity are analytically propagated to the next time.

***Proposition*** ***1.*** *Suppose that the posterior intensity* $v_{k-1}$ *at time* k−1 *is a Gaussian form:*

(6) $$v_{k-1}\left(x|Z^{k-1}\right)=\sum_{i=1}^{J_{k-1}}w_{k-1}^{\left(i\right)}N\left(x;m_{k-1}^{\left(i\right)},P_{k-1}^{\left(i\right)}\right)$$

*Then, for a linear Gaussian multi-target model, the predicted intensity* $v_{k|k-1}$ *at time* k *is also a Gaussian mixture, and is given by:*

(7) $$v_{k|k-1}\left(x\right)=p_{S,k}\sum_{i=1}^{J_{k-1}}w_{k-1}^{\left(i\right)}N\left(x;m_{S,k|k-1}^{\left(i\right)},P_{S,k|k-1}^{\left(i\right)}\right)+\gamma_{k}\left(x\right)$$

*where* $\gamma_{k}\left(x\right)$ *is given in Equation (5). The predicted mean* $m_{S,k|k-1}^{\left(i\right)}$ *and covariance* $P_{S,k|k-1}^{\left(i\right)}$ *are given by the standard Kalman prediction equation, whose pseudo-function is:*

(8) $$\left[m_{S,k|k-1}^{\left(i\right)},P_{S,k|k-1}^{\left(i\right)}\right]=KF_{p}\left(m_{k-1}^{\left(i\right)},P_{k-1}^{\left(i\right)},F_{k-1},Q_{k-1}\right)$$

***Proposition*** ***2.*** *Suppose that the predicted intensity* $v_{k|k-1}$ *for time* k *is a Gaussian form:*

(9) $$v_{k|k-1}\left(x\right)=\sum_{i=1}^{J_{k-1|k}}w_{k|k-1}^{\left(i\right)}N\left(x;m_{k|k-1}^{\left(i\right)},P_{k|k-1}^{\left(i\right)}\right)$$

*and we collect a new measurement set* $Z_{k}=\left\{z_{1},\cdots ,z_{m_{k}}\right\}$ *with* $\left|Z_{k}\right|=m_{k}$ *at time step* k *. Then, for a linear Gaussian multi-target model, the posterior intensity* $v_{k}$ *at time* k *is given by:*

(10) $$v_{k|k}\left(x|Z^{k}\right)=\left(1-p_{D,k}\right)D_{k|k-1}\left(x\right)+\sum_{i=1}^{J_{k+1|k}}\sum_{j=1}^{m_{k}}w_{k|k}^{\left(i,j\right)}N\left(x;m_{k|k}^{\left(i,j\right)},P_{k|k}^{\left(i,j\right)}\right)$$

*where:*

(11) $$w_{k|k}^{\left(i,j\right)}=\frac{p_{D,k}w_{k|k-1}^{\left(i\right)}q_{k|k-1}^{\left(i,j\right)}}{\kappa \left(z_{j}\right)+p_{D,k}\sum_{l=1}^{J_{k+1|k}}w_{k|k-1}^{\left(l\right)}q_{k|k-1}^{\left(i,j\right)}}$$

(12) $$\left[m_{k|k}^{\left(i,j\right)},P_{k|k}^{\left(i,j\right)},q_{k|k-1}^{\left(i,j\right)}\right]=KF_{E}\left(m_{k|k-1}^{\left(i\right)},P_{k|k-1}^{\left(i\right)},z_{j},H_{k},R_{k}\right)$$

*where Equation (12) is the Kalman filter estimation pseudo-function, and* $q_{k|k-1}^{\left(i,j\right)}$ *is a probability density function with mean* $H_{k}m_{k|k-1}^{\left(i\right)}$ *and covariance* $H_{k}P_{k|k-1}^{\left(i\right)}H_{k}^{T}+R_{k}$ *for measurement prediction.*

## 3. GMPHDF Incorporating Map Information

This section mainly describes two types of GMPHD filter, incorporating map information and a road segment switching algorithm. Specifically, the DPN-GMPHD and SC-GMPHD filters will be presented in this section. From refs. [37,45] we can see that a given road through a real road network is described by nodes and edges in digital roadmaps, where nodes indicate that a road changes quality and edges represent individual road segments. The process of using roadmap information to assist ground target tracking can be divided into two steps. The first is to make use of road information to constrain the state of the target moving on each individual road segment. The second is to use a road segment switching algorithm to perform ground target tracking on a road. In this section, the first step is described in the first two subsections, and the road segment switching algorithm is subsequently given in Section 3.3.

### 3.1. DPN-GMPHD Filter

**Figure 1:**
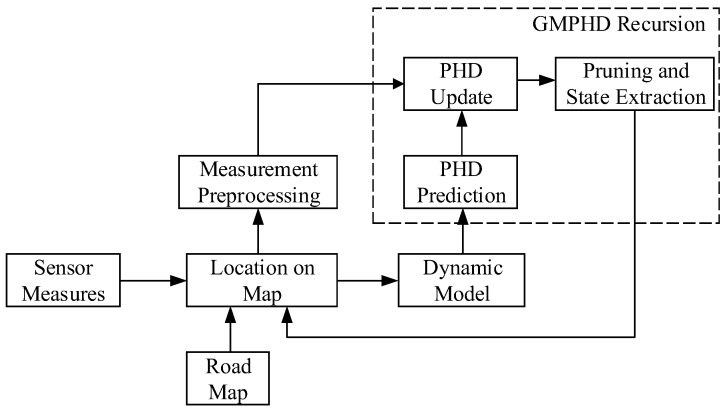
Framework of the directional process noise Gaussian mixture probability hypothesis density (DPN-GMPHD) filter.

The performance of the DPN-GMPHD filter is improved by modifying the process noise covariance of the dynamic model using roadmap information. The basic framework of the filter is shown in Figure 1.

In the DPN-GMPHD recursion, the concept of directional process noise (DPN) is adopted to handle the motion along a road. Typically, the off-road target can move in any direction, so the motion uncertainties in both the X and Y directions are equal. For on-road targets, the motion uncertainty along the road is more than orthogonal to it because of the road constraint. In the off-road target motion model, $v_{x}$ and $v_{y}$ are process noise components in the X and Y directions, respectively, and $\sigma_{x}^{2}$ and $\sigma_{y}^{2}$ are the corresponding process noise variances. In the on-road target motion model, $v_{a}$ and $v_{o}$ are process noise components along and orthogonal to the road, respectively, and $\sigma_{a}^{2}$ and $\sigma_{o}^{2}$ are the corresponding process noise variances. It is known from the previous discussion that $\sigma_{x}^{2}=\sigma_{y}^{2}$ and $\sigma_{a}^{2}\gg \sigma_{o}^{2}$.

Since filter recursion is performed in the Cartesian ground coordinates, the process noise variances in the on-road target motion model, $\sigma_{a}^{2}$ and $\sigma_{o}^{2}$, need to be converted into a covariance matrix, $q_{xy}$, in that frame. This is done via [3,46,47]:

(13) $$q_{xy}=\left[\begin{array}{cc} -\cos\varphi & \sin\varphi \\ \sin\varphi & \cos\varphi \end{array}\right]\left[\begin{array}{cc} \sigma_{o}^{2} & 0\\ 0 & \sigma_{a}^{2} \end{array}\right]\left[\begin{array}{cc} -\cos\varphi & \sin\varphi \\ \sin\varphi & \cos\varphi \end{array}\right]\;$$

where φ is the angle between the road and the north direction. When the target state is represented as $x=\left(x,\dot{x},y,\dot{y}\right)$ in Cartesian coordinates, the corresponding process noise covariance matrix $Q_{xy}$ is calculated as:

(14) $$Q_{xy}=Bq_{xy}B^{\prime }\;$$

(15) B=I2×2⊗[T22T] 

where B denotes the process noise allocation matrix, T is the sampling interval, $I_{2\times 2}$ is the second-order identity matrix, and ⊗ denotes the Kronecker product.

With the above directional process noise covariance matrix $Q_{xy}$, the DPN-GMPHD filter can directly apply.

### 3.2. SC-GMPHD Filter

**Figure 2:**
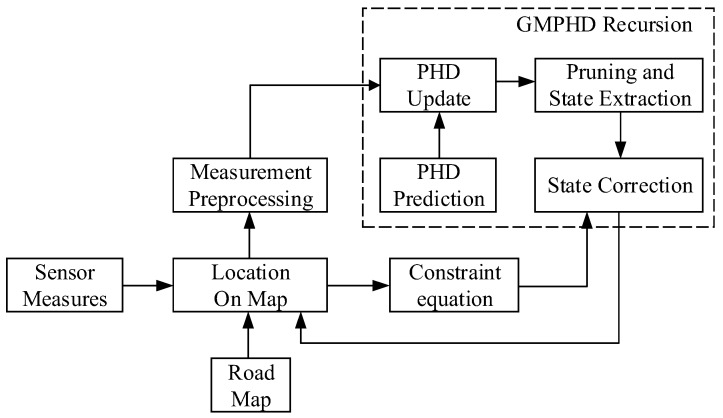
Framework of the state constraint (SC)-GMPHD filter.

In the SC-GMPHD filter, roadmap information is firstly exploited to establish the constraint relationship among the position parameters in the target state, and then this constraint relationship is used to modify the result of the target state estimation. The basic framework of the SC-GMPHD filter is shown in Figure 2.

The main steps of the SC-GMPHD filter are described in detail below. The first step involves employing the roadmap to establish a basic state constraint. In Cartesian coordinates, assume that the target state is $x_{g,k}={\left[x_{g,k},{\dot{x}}_{g,k},y_{g,k},{\dot{y}}_{g,k}\right]}^{T}$ at time step k, and the coordinates of the start and end positions of a road segment are $s_{1}=\left(x_{1},y_{1}\right)$ and $s_{2}=\left(x_{2},y_{2}\right)$, respectively. Then the angle between the along-road direction and Y direction (north direction), φ, is given by:

(16) $$\varphi =\mathrm{atan}\left(\frac{x_{2}-x_{1}}{y_{2}-y_{1}}\right)$$

Assume that when a target is moving on this road segment, the following two constraints must be satisfied simultaneously:

(1) The trajectory of the target is a straight line, and the target position is on the center line of the road segment (the connection between the start and the end of the road), i.e.:
  (17) $$\frac{x_{g}-x_{1}}{y_{g}-y_{1}}=\frac{x_{2}-x_{1}}{y_{2}-y_{1}}=\tan\varphi \;$$
(2) The direction of the target velocity is parallel to the direction of the road segment, i.e.:
  (18) $$\frac{{\dot{x}}_{g}}{{\dot{y}}_{g}}=\frac{x_{2}-x_{1}}{y_{2}-y_{1}}=\tan\varphi \;$$

The above two constraints can be expressed as an equation set:

(19) $$\left\{ \begin{array}{l} x_{g}-y_{g}\tan\varphi =x_{1}-y_{1}\tan\varphi \\ {\dot{x}}_{g}-{\dot{y}}_{g}\tan\varphi =0 \end{array}\right.\;$$

The above equations can also be expressed as:

(20) $$\left[\begin{array}{cccc} 1 & 0 & -\tan\varphi & 0\\ 0 & 1 & 0 & -\tan\varphi \end{array}\right]\left[\begin{array}{c} x_{g}\\ {\dot{x}}_{g}\\ y_{g}\\ {\dot{y}}_{g} \end{array}\right]=\left[\begin{array}{c} x_{1}-y_{1}\tan\varphi \\ 0 \end{array}\right]\;$$

Let:

(21) $$D=\left[\begin{array}{cccc} 1 & 0 & -\tan\varphi & 0\\ 0 & 1 & 0 & -\tan\varphi \end{array}\right],\;d=\left[\begin{array}{c} x_{1}-y_{1}\tan\varphi \\ 0 \end{array}\right]$$

Then, Equation (20) can also be given by:

(22) $$Dx_{g}=d$$

Referring to the method proposed in Refs. [48,49,50], the target state estimation of the filter can be modified by the above constraints.

Based on the above discussion, the recursive process of the SC-GMPHD filter is given as shown below. Assume that the *i*th Gaussian component of the SC-GMPHD filter at time *k* is $\left\{w_{k|k-1}^{\left(i\right)},m_{k|k-1}^{\left(i\right)},P_{k|k-1}^{\left(i\right)}\right\}$. Then, the corresponding update component for measurement is $\left\{w_{k}^{\left(i\right)}\left(z\right),m_{k|k}^{\left(i\right)}\left(z\right),P_{k}^{\left(i\right)}\left(z\right)\right\}$. Furthermore, the constraint of Equation (22) is used to modify the mean $m_{k|k}^{\left(i\right)}\left(z\right)$ of the updated Gaussian component, and the modified equation is given by:

(23) $${\tilde{m}}_{k|k}^{\left(i\right)}\left(z\right)=m_{k|k}^{\left(i\right)}\left(z\right)-P_{k}^{\left(i\right)}\left(z\right)D^{T}{\left[DP_{k}^{\left(i\right)}\left(z\right)D^{T}\right]}^{-1}\left(Dm_{k|k}^{\left(i\right)}\left(z\right)-d\right)$$

With the above modified equation, the SC-GMPHD filter can be directly applied.

### 3.3. Road Segment Switching Algorithm

**Figure 3:**
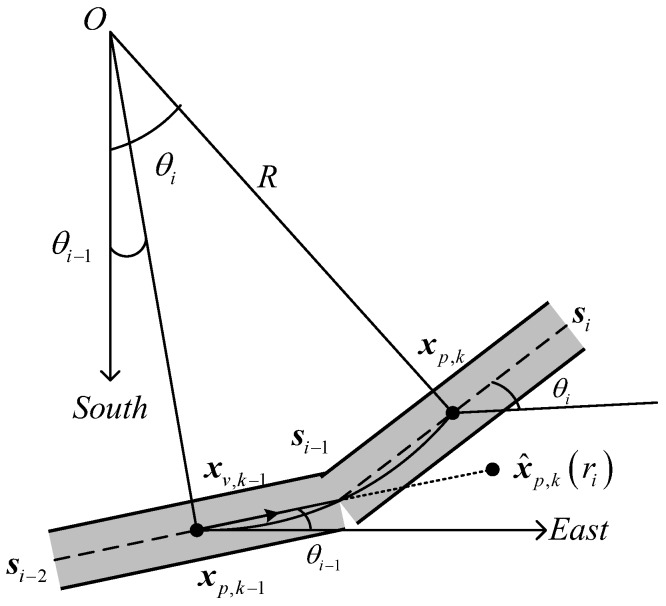
Schematic diagram of a curved road in a digital roadmap.

As shown in Figure 3, when a target moves along a curved road with radius R, the angle θ between the road-parallel and east directions will change over time. Therefore, in the following we specifically discuss the roadmap fusion method for the filters presented in this paper when the target is moving on a curved road. Figure 3 also shows that a curved road is approximated by a set of straight road segments in a digital vector map. In the previous three subsections, we analyzed the situation of the target moving on each road segment. In the following, we analyze the situation when the target moves from one road segment to the next.

Let r denote road segment information, which can be used as an input parameter for the filters proposed in this paper. For a straight road, r is a constant. However, for a curved road, r is a time-dependent parameter that can be expressed as r(t). The following mainly discusses the change of r(t) when the target moves from road segment i−1 to i during a time interval $\left(t_{k-1},t_{k}\right)$.

If the target is located on road segment i−1 at time k−1, as shown in Figure 3, the target position and velocity are represented as $x_{p,k-1}$ and $x_{v,k-1}$, respectively. As can be seen from Figure 3, the real target position is $x_{p,k}$ at time k. In the time interval $\left(t_{k-1},t_{k}\right)$, given the velocity estimation of the target on road segment i−1 at time k−1, the position of the target can be predicted as ${\hat{x}}_{p,k}\left(r_{i}\right)$ at time k, as shown in Figure 3. This is obviously inconsistent with real-life situations. Therefore, in order to use the proposed filters to track the target on a curved road, it is necessary to determine whether the road segment on which the target is located will change at the next time step. The case when $\left\|{\hat{x}}_{p,k|k-1}\left(r_{i}\right)-x_{p,k-1}\right\|-\left\|s_{i-1}-x_{p,k-1}\right\|>0$ means that the road segment on which the target is located will change at the next time step. In this case, the target state prediction in the time interval $\left(t_{k-1},t_{k}\right]$ requires segmentation processing according to the road information. In the case that that the target’s velocity is constant, the times taken for the target to pass along segments i−1 and i are $\Delta t_{k,i-1}=\left\|s_{i-1}-x_{p,k-1}\right\|/\left|x_{v,k-1}\right|$ and $\Delta t_{k,i}=t_{k}-t_{k-1}-\Delta t_{k,i-1}$, respectively. As a result, the road information used for predicting the target state in the time interval $\left(t_{k-1},\Delta t_{k,i-1}\right]$ is $r_{i-1}$, but the road information used for state prediction and update in $\left(\Delta t_{k,i-1},t_{k}\right]$ is $r_{i}$.

When the road segment changes from $r_{i-1}$ to $r_{i}$, the velocity of the target changes from $x_{v,k-1}$ to:

(24) $$x_{v,t_{k-1}+\Delta t_{k,i-1},i}=\left|x_{v,k-1}\right|{\left(\cos\theta_{i-1},\cos\theta_{i}\right)}^{T}\;$$

where $x_{v,t_{k-1}+\Delta t_{k,i-1},i}$ denotes the velocity component of the target on road segment i at time $t_{k-1}+\Delta t_{k,i-1}$.

### 3.4. Simulation Results

This section presents two simulation examples to demonstrate the performance of the filters proposed in this paper. Firstly, two single-target tracking simulation scenarios are considered, in which a GMTI radar is applied to detect the targets. Secondly, the proposed filtering algorithms are used to process the measurements made by the GMTI radar. Finally, the evaluations made using the optimal subpattern assignment (OSPA) distance and root mean square error (RMSR) are used to compare the performances of the filters comprehensively. The definitions of these two performance evaluation criteria are given below. The RMSR is given by:

(25) $$RMSE=\sqrt{\frac{1}{N}\sum_{i=1}^{N}{\left(X_{filter,i}-X_{truth,i}\right)}^{2}}$$

The OSPA distance, an error distance used to measure the degree of difference between sets [51], is defined as:

(26) $${\bar{d}}_{p}^{\left(c\right)}\left(X,Y\right)=\left\{ \begin{array}{ll} 0, & m=n=0\\ {\left(\frac{1}{n}\left(\underset{\pi \in \Pi_{n}}{\min}\sum_{i=1}^{m}d^{\left(c\right)}{\left(x_{i},y_{\pi \left(i\right)}\right)}^{p}+c^{p}\left(n-m\right)\right)\right)}^{\frac{1}{p}}, & m\leq n\\ {\bar{d}}_{p}^{\left(c\right)}\left(Y,X\right), & m>n \end{array}\right.$$

where $d^{\left(c\right)}\left(x,y\right):=\min\left(c,\left\|x-y\right\|\right)$, c is the cutoff distance and $\Pi_{n}$ denotes the set of permutations on {1,2,⋯,n} for any $n\in {\mathbb{N}}_{+}$.

**Figure 4:**
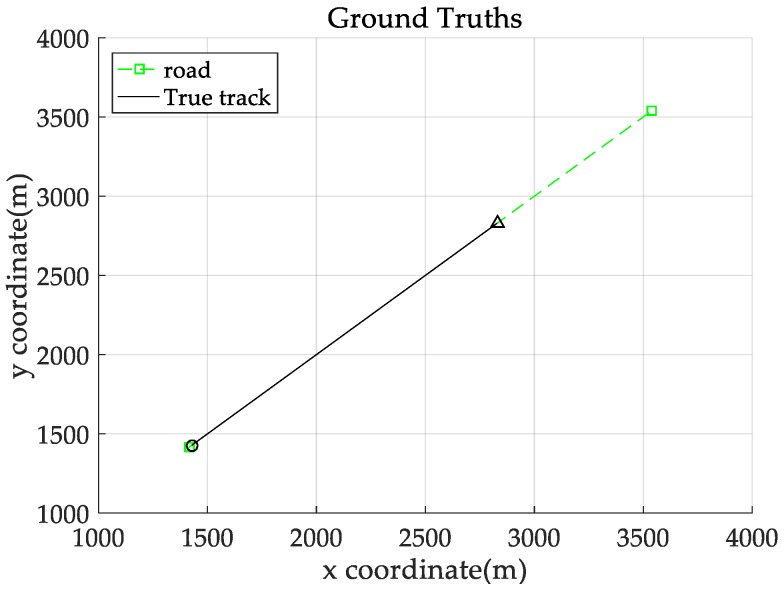
Road information (green dotted line) and target real trajectory (black solid line). “○”: location at which the target starts; “△”: location at which the target stops.

***Example*** ***1***: *Consider a 2-D plane single-target tracking scenario, as shown in Figure 4. In this scenario, there is a single target moving along a straight road at a constant speed of 20 m/s. The starting position of this target is indicated by the symbol “○”, and the stopping position is indicated by “△”. The start and end coordinates of the straight road in this scenario are (1415, 1415) and (3536, 3536), respectively, as shown by the dotted green line.*

**Figure 5:**
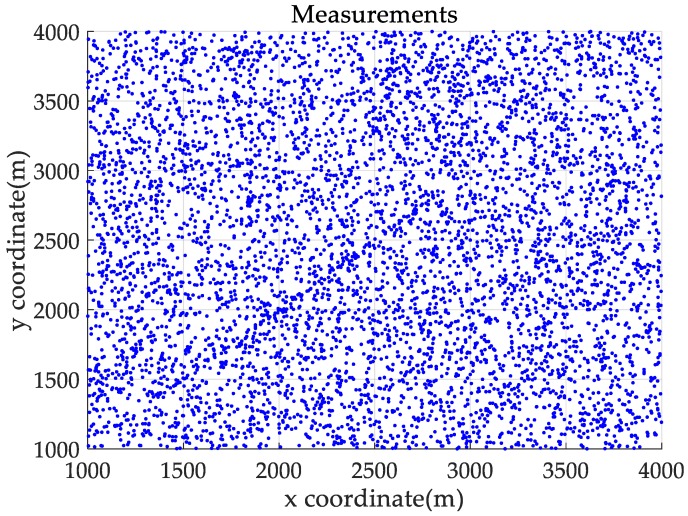
Measurements.

The application of the GMTI radar output relies on the correct modeling of the GMTI radar properties. The natural coordinate system for the description of the GMTI radar measurements are polar coordinates. For simplicity, we assume that the sensor adopts ground Cartesian coordinates, in order to avoid nonlinear transformations between the two coordinate systems during data processing. The original measurement components from the GMTI radar are range, azimuth, and potential range rate. In the present study, measurements of range rate are not taken into account [1]. Based on the above assumptions, the measurement made by the sensor is the target position in Cartesian coordinates, i.e., the measurement vector z of a single target is denoted as $z={\left(x,y\right)}^{T}$. The measurement error is a Gaussian random vector with mean 0 and covariance R, where $R=\mathrm{diag}\left(\sigma_{x}^{2},\sigma_{y}^{2}\right)$, and $\sigma_{x}=10\;m$ and $\sigma_{y}=10\;m$ are the standard deviations of the measurement error in the X and Y directions, respectively. The detection probability is $p_{D}=0.9$. Clutter follows a Poisson RFS with intensity $\kappa \left(z\right)=\lambda_{c}Vu\left(z\right)$, where $\lambda_{c}$ denotes the clutter intensity, $V=1.6\times 10^{7}m^{2}$ is the “volume” of the surveillance region and u(⋅) is a uniform probability density over the surveillance region. Figure 5 shows a plot of the true trajectory with cluttered measurements against time, when the clutter intensity is $\lambda_{c}=3.75\times 10^{-6}\;m^{-2}$ and the sampling time (data length) is K=100.

The kinematic state of the target is defined as:

(27) $$x={\left(x,\dot{x},y,\dot{y}\right)}^{T}$$

where (x,y) denotes the position of the target in Cartesian coordinates and $\left(\dot{x},\dot{y}\right)$ is the corresponding velocity information. The Measuring matrix H is given by:

(28) $$H=I_{2\times 2}⊗\left(\begin{array}{cc} 1 & 0 \end{array}\right)$$

where $I_{2\times 2}$ denotes a second-order identity matrix and ⊗ denotes the Kronecker product.

For the standard GMPHD filter, the state transition matrix F and the process noise covariance matrix Q are:

(29) $$F=I_{2\times 2}⊗\left(\begin{array}{cc} 1 & T\\ 0 & 1 \end{array}\right)\;$$

(30) $$Q=q^{2}BB^{T}$$

(31) $$B=I_{2\times 2}⊗\left(\begin{array}{c} \frac{1}{2}T^{2}\\ T \end{array}\right)$$

where q=2 and T=1 s.

For the CT-GMPHD filter, the state transition matrix $F_{r}$ and the process noise covariance matrix $Q_{r}$ in road coordinates, are given by:

(32) $$F_{r}=\left(\begin{array}{cc} 1 & T\\ 0 & 1 \end{array}\right)$$

(33) $$Q=q^{2}B_{0}B_{0}^{T}$$

(34) $$B_{0}=\left(\begin{array}{c} \frac{1}{2}T^{2}\\ T \end{array}\right)$$

where q=2.

For the DPN-GMPHD filter, the state transition matrix $F_{d}$ is given by Equation (29), and the process noise covariance matrix $Q_{d}$ is given by:

(35) $$Q_{d}=Bq_{d}B^{T}$$

(36) $$q_{d}=Gq_{r}G$$

(37) $$G=\left(\begin{array}{cc} -\cos\varphi & \sin\varphi \\ \sin\varphi & \cos\varphi \end{array}\right)$$

(38) $$q_{r}=\mathrm{diag}\left(\sigma_{o}^{2},\sigma_{a}^{2}\right)\;$$

where φ=π/4, $\sigma_{a}^{2}=q^{2}\sin\varphi +q^{2}\cos\varphi$, and $\sigma_{o}^{2}$ is determined by the width of the road. In this paper $\sigma_{o}^{2}=0.2911q^{2}$.

For the SC-GMPHD filter, the state transition matrix and the process noise covariance matrix are the same as the corresponding parameters of the standard GMPHD filter. The pruning parameters of all filters used in this paper are as follows: truncation threshold: $1\times 10^{-5}$; merging threshold: 4 m; and the maximum allowable number of Gaussian terms is 100.

Based on the above parameters, a GMPHD filter without roadmap information, and three GMPHD filters with roadmap information, are explored to estimate the target state using the measurements shown in Figure 5. Additionally, the OSPA distance (average on time), with parameters p=1 and c=100, and the RMSE, are used to measure the performance of the filters. Table 1 gives a comparison of the filtering performance of the four filters. Overall, the performance of filters with roadmap information is better than that of filters without roadmap information. The CT-GMPHD and SC-GMPHD filters obtain similar estimation results for the target state, and the position estimates are entirely confined to the road. Although the result of the position estimation for the DPN-GMPHD filter is not entirely confined to the road, it is very close to it, and its OSPA value is smaller than that of the standard GMPHD filter.

**Figure 6:**
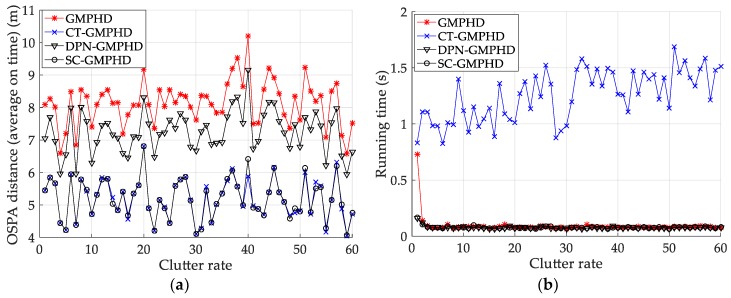
Comparison of tracking performance versus clutter rate: (**a**) average optimal subpattern assignment (OSPA) of the tracking algorithms; (**b**) running time of the tracking algorithms.

We subsequently analyze the estimation performance of the proposed filters when the detection probability and clutter density are different. Figure 6 and Table 2 show the performance (OSPA distance and simulation running time) of each filter in different clutter environments, with a detection probability of 0.9. From Figure 6a and Table 2, it can be seen that the accuracy of the target state estimated by the GMPHD filter incorporating roadmap information is significantly higher than that of the GMPHD filter without road information. The filtering accuracy of the CT-GMPHD filter approaches that of the SC-GMPHD filter and is slightly better than that of the DPN-GMPHD filter. From Figure 6b and Table 2, it can be seen that the running time of the CT-GMPHD filter is more than 15 times longer than that of the three other filters. Moreover, the running times of the two filters proposed in this paper are very similar, and lower than that of the standard GMPHD filter.

**Figure 7:**
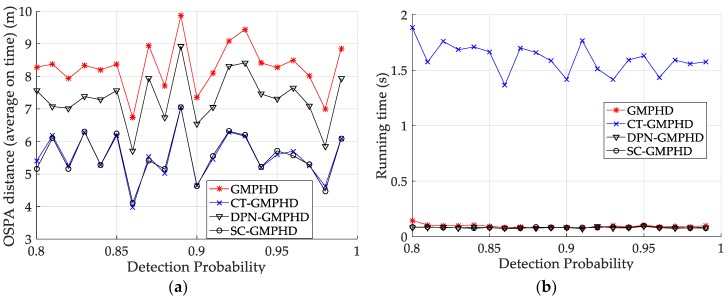
Tracking performance of the filters versus detection probability: (**a**) average OSPA of the filters; (**b**) running time of the filters.

Figure 7 and Table 3 show the tracking performance (OSPA distance and simulation running time) of the filters versus detection probability, for a clutter rate of 60. The results are similar to the tracking performances shown in Figure 6 and Table 2.

**Figure 8:**
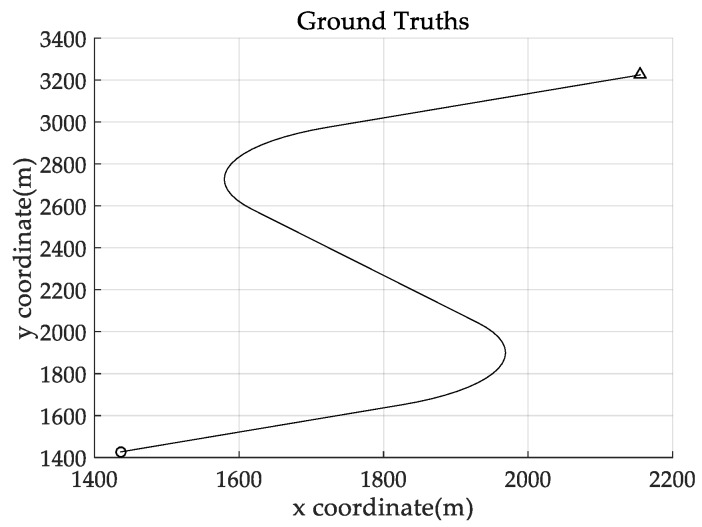
True trajectory of the target moving on a curved road: “○”: location at which the target starts; “△”: location at which the target stops.

***Example*** ***2***: *In this example, we evaluate the performance of the proposed filters using a curved road tracking scenario, as shown in Figure 8. This scenario involves a single target moving along a curved road at a constant speed of 20 m/s. The target starts at* *t* *= 1 s, with the initial state (1436, 21.7, 1427, 12.5)*^T^*, and stops at t = 100 s. From t = 1 s to t = 19 s, the target has constant velocity along a straight road segment, and from t = 20 s to t = 37 s, it undergoes constant turn motion with a turn rate ω = 0.0873 rad/s. The target then turns onto another straight road segment, and moves along it with constant velocity from t = 38 s to t = 62 s. From t = 63 s to t = 80 s, the target undergoes constant turn motion with a turn rate ω = −0.0873 rad/s, before turning onto a straight road. From t = 81 s to t = 100 s the target has a constant velocity.*

**Figure 9:**
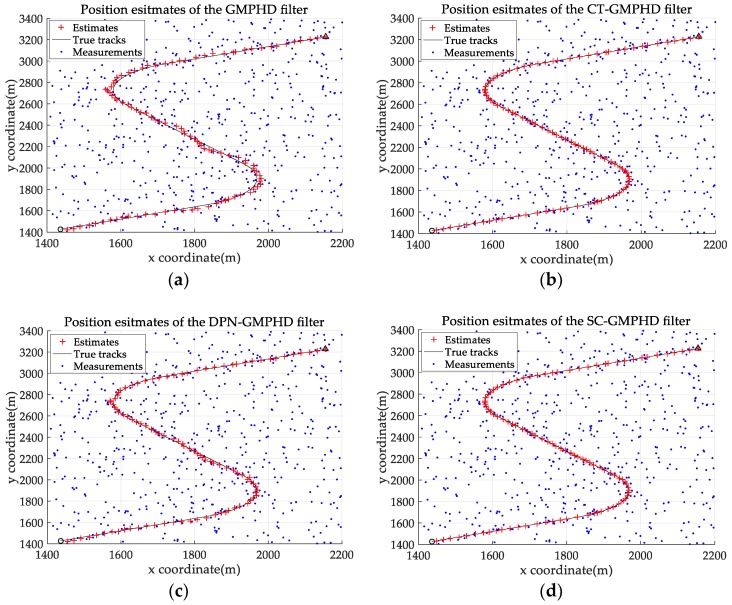
Filtering performance comparison of four filters for a target on a curved road: (**a**) standard GMPHD filter; (**b**) CT-GMPHD filter; (**c**) DPN-GMPHD filter; (**d**) SC-GMPHD filter.

**Figure 10:**
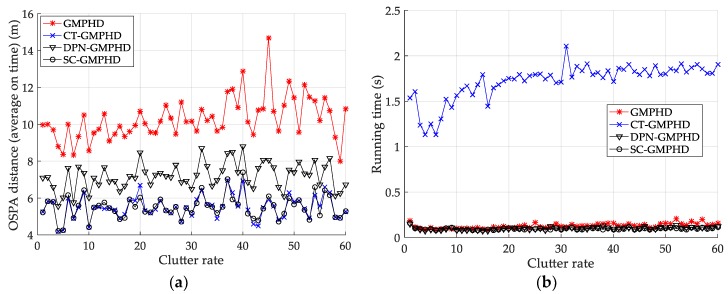
Comparison of tracking performance versus clutter rate for a target on a curved road: (**a**) average OSPA of the tracking algorithms; (**b**) running time of the tracking algorithms.

**Figure 11:**
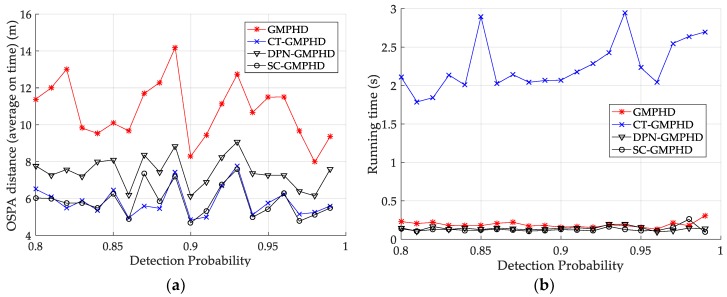
Tracking performance of the filters versus the detection probability for a target on a curved road: (**a**) average OSPA of the filters; (**b**) running time of the filters.

The experimental settings are as the same as those in Example 1. The simulation results for a curved road tracking scenario are shown in Figure 9, Figure 10 and Figure 11 and Table 4, Table 5 and Table 6. Figure 9 and Table 4 show the comparison of the filtering performance of four filters for a target on a curved road, with a detection probability of 0.9 and a clutter rate of 6. Figure 10 and Table 5 depict the performance (OSPA distance and simulation running time) of each filter in different clutter environments when the target is on a curved road, with a detection probability of 0.9. Figure 11 and Table 6 give the tracking performance of the filters versus the detection probability for the target on a curved road.

From the above simulation results, it can be seen that: (i) filters with roadmap information outperform the filters without roadmap information; (ii) the filtering accuracy of the CT-GMPHD filter is similar to that of the SC-GMPHD filter, but slightly better than that of the DPN-GMPHD filter; and (iii) the running time of the CT-GMPHD filter is more than 11 times longer than that of the three other filters. In addition, compared to the straight road tracking scenario, the filtering accuracy of the target in the curved road tracking scenario is not reduced, while the running time increases.

In summary, the proposed filtering algorithm can improve both the filtering accuracy and computational efficiency simultaneously. Furthermore, the proposed filter is able to better adapt to tracking requirements in different clutter and detection environments.

It should be noted that the implementation of the filters proposed in this paper is based on the basic assumptions of the GMPHD filter [29]. In practical applications, if the application environment does not satisfy these assumptions, the accuracy of the target state estimation will be affected to some extent. The performance of the filters proposed in this paper will additionally be affected by roadmap accuracy.

## 4. Comparison of the CPHD and LMB Filters

This section describes a road-constrained method combined with DPN (proposed in Section 3.1) and SC (proposed in Section 3.2) methods. A DPN method and an SC method are applied to both the prediction and update steps of the same filter. The road constraint method is then applied to the PHD, CPHD, and LMB filters. Finally, the performance of the three filtering algorithms is compared and analyzed using a ground target tracking simulation scenario.

### 4.1. The CPHD and LMB Filters

Since the PHD filter is a first-order statistical moment for multiple target densities, high-order information is lost, resulting in an unstable target number estimate in a low SNR environment. Conversely, the PHD filter uses only one parameter to propagate the cardinality information of the target, and the unknown cardinality distribution is approximated as a Poisson distribution. Since the mean and covariance of the Poisson distribution are equal, when the number of targets is relatively large, the covariance of the target number estimate of the PHD filter will be correspondingly larger. In response to the above problem, Mahler relaxed the target number mean to obey the Poisson distribution hypothesis and derived the CPHD filter [15]. The CPHD filter can simultaneously propagate the multi-target intensity function and the cardinality distribution information of the target. Therefore, the CPHD filter can obtain a more accurate target number estimate than the PHD filter [15,16]. A previous study [26] gives the GM implementation of the CPHD filter under the assumption of a linear Gaussian model [17].

In addition to the PHD and CPHD filters, Mahler also proposed a multi-target multi-Bernoulli (MeMBer) filter to approximate multi-target Bayesian filtering. However, the MeMBer filter has a significant bias in the number of targets [10]. To reduce the cardinality bias, the cardinality balanced MeMBer (CBMeMBer) filter was proposed by B.T. VO [24]. In this paper, the CBMeMBer filter will hereafter be abbreviated as the “MB filter”. Previous studies [21,22] have proposed a generalized LMB filter to estimate the trajectory of multiple targets.

### 4.2. Simulation Results

In this subsection, we evaluate the performance of the PHD, CPHD, and LMB filters for ground target tracking in the curved road tracking scenario shown in Figure 8. Before this, the road-constrained method is applied to the above three filters. The PHD, CPHD, and LMB filtering algorithms using the road-constrained method are hereafter referred to as the RC-PHD, RC-CPHD, and RC-LMB filters. All filters are implemented using the Gaussian mixture method.

Table 7 gives a comparison of the filtering performance of the three filters when the detection probability and clutter are equal to 0.95 and 6, respectively. It can be seen that the RC-PHD filter, while being the cheapest computationally, gives a slightly worse performance than the RC-CPHD and RC-LMB filters.

**Figure 12:**
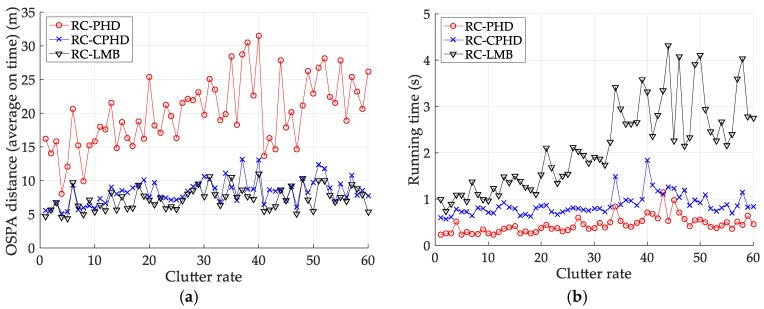
Comparison of tracking performance versus clutter rate for a target on a curved road: (**a**) average OSPA of the tracking algorithms; (**b**) running time of the tracking algorithms.

**Figure 13:**
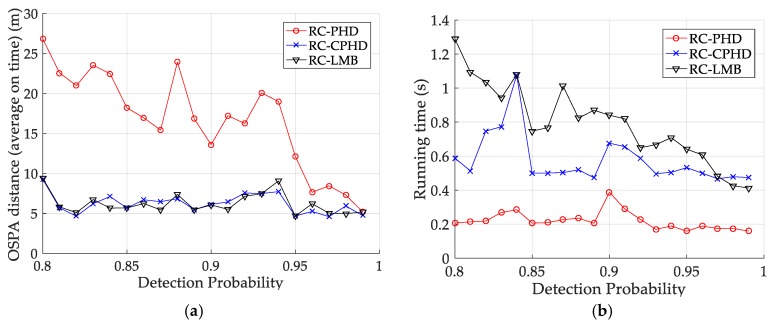
Tracking performance of the filters versus detection probability for a target on curved road: (**a**) average OSPA of the filters (**b**) running time of the filters.

Figure 12 and Table 8 depict the performance (OSPA distance and simulation running time) of each filter for different clutter environments, where the target is on a curved road and the detection probability is equal to 0.95. Figure 13 and Table 9 give the tracking performance of the filters versus the detection probability for a target on a curved road when the clutter rate is equal to 6. From the above simulation results, it can be seen that: (i) the filtering accuracy of the RC-CPHD filter is similar to that of the RC-LMB filter, and slightly better than that of the RC-PHD filter; (ii) the running time of the RC-CPHD filter is slightly shorter than that of the RC-LMB filter, but longer than that of the RC-PHD filter.

In summary, the target estimation accuracy of the RC-PHD filter is slightly worse than that of the RC-CPHD and RC-LMB filters, although it is computationally the cheapest.

## 5. Conclusions

In this paper, the GMPHD filter is applied to the problem of tracking ground targets. DPN and state constraints (SC) are used to integrate a digital roadmap into the GMPHD filter. In the DPN-GMPHD recursion, the concept of DPN is employed to analyze motion along a road. For on-road targets, the motion uncertainty along the road is more than that in the orthogonal direction, as a result of the road constraint. For the SC-GMPHD filter, the road constraint is considered to modify the result of the target state estimation at each time step. To analyze the performance of two filters proposed in this paper, two simulation scenarios of a target moving along a straight road and a curved road are presented. The simulation results show that, for tracking a ground target along the single road, the filtering algorithms proposed in this paper significantly improve the tracking accuracy and computational efficiency compared with the standard GMPHD and CT-GMPHD filters, when the clutter rate and detection probability are uncertain. Although the two filters proposed in this paper have obvious advantages over existing filtering algorithms, they also have several limitations. Firstly, it should be noted that the two filters are only suitable for tracking targets on a single road. In reality, however, there are typically multiple targets moving in a road network, even where there is no roadmap information. Therefore, it is necessary to introduce a multi-model approach to allow the tracking of multiple ground targets moving on different roads. Furthermore, we assume that the sensor has a linear Gaussian measurement model. In practice, it is necessary to transform the measurement information in the sensor coordinate system into a Cartesian coordinate system through measurement conversion. These limitations necessitate further research. In addition to the PHD filter, the proposed methods are also used with the CPHD and LMB filters. A curved road tracking scenario is used to evaluate the performance of these three filter types. The simulation results show that the target estimation accuracy of the RC-PHD filter is slightly worse than that of the RC-CPHD and RC-LMB filters, but that it is computationally the cheapest.

## Figures and Tables

**Table 1:** Filtering performance comparison of four filters.

Filter Type	Filter Estimates	Fractionated Gain	OSPA Distance	RMSE
GMPHD	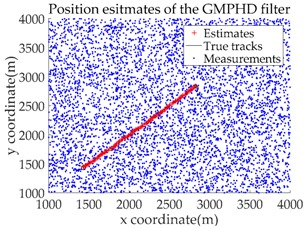	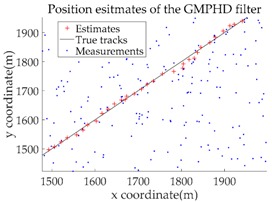	8.8919	7.1376 6.2848
CT-GMPHD	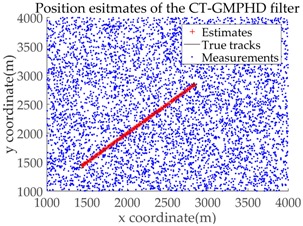	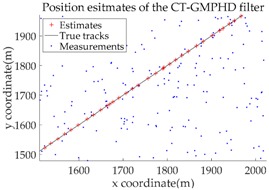	6.0831	4.8847 4.8847
DPN-GMPHD	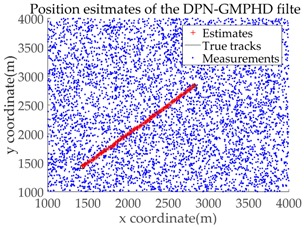	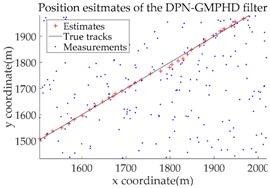	7.4390	6.4401 5.9297
SC-GMPHD	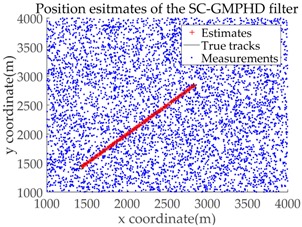	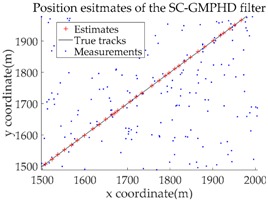	6.0575	4.8371 4.8371

**Table 2:** Average OSPA distance and running time of different filters with different clutter rates, for a detection probability of 0.9.

Filter Type	Average OSPA Distance (m)	Average Running Time (s)
GMPHD	8.1407	0.0613
CT-GMPHD	5.1985	1.0868
DPN-GMPHD	7.2513	0.0599
SC-GMPHD	5.2027	0.0603

**Table 3:** Average OSPA distances and running times of different filters versus detection probability, for a clutter rate of 60.

Filter Type	Average OSPA Distance (m)	Average Running Time (s)
GMPHD	8.2889	0.0664
CT-GMPHD	5.5590	1.2926
DPN-GMPHD	7.3415	0.0618
SC-GMPHD	5.5516	0.0619

**Table 4:** Average OSPA distance and RMSE comparison of the four filters for a target on a curved road.

Filter Type	Average OSPA Distance	RMSE
GMPHD	10.7596	9.5748, 7.3602
CT-GMPHD	5.9806	4.5927, 5.5801
DPN-GMPHD	8.0298	6.6143, 6.2683
SC-GMPHD	5.9927	4.5898, 5.5882

**Table 5:** Average OSPA distance and running time of the different filters with different clutter rates when the detection probability is equal to 0.9.

Filter Type	Average OSPA Distance (m)	Average Running Time (s)
GMPHD	10.2843	0.0966
CT-GMPHD	5.4990	1.3550
DPN-GMPHD	7.1933	0.0742
SC-GMPHD	5.5016	0.0718

**Table 6:** Average OSPA distance and running time of the different filters versus the detection probability when the clutter rate is equal to 60.

Filter Type	Average OSPA Distance (m)	Average Running Time (s)
GMPHD	10.7964	0.1369
CT-GMPHD	5.8325	1.5364
DPN-GMPHD	7.4537	0.0956
SC-GMPHD	5.8517	0.0884

**Table 7:** Filtering performance comparison of three filters.

Filter Type	Filter Estimates	OSPA Distance	Average OSPA	Running Time
RC-PHD	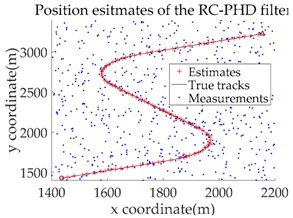	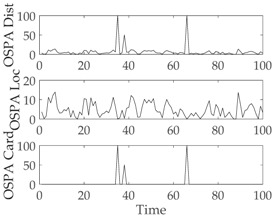	7.2722	0.7821
RC-CPHD	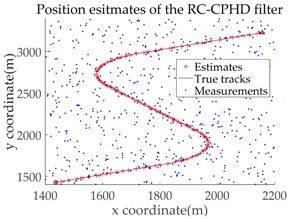	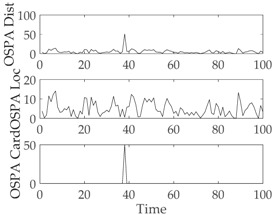	5.4188	1.2313
RC-LMB	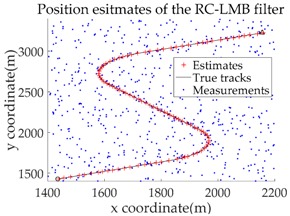	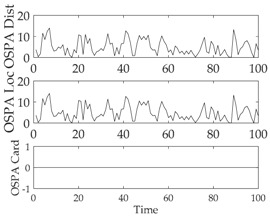	4.9550	1.4982

**Table 8:** Average OSPA distance and running time of different filters with different clutter rates when the detection probability is equal to 0.95.

Filter Type	Average OSPA Distance (m)	Average Running Time (s)
RC-PHD	20.1743	0.2941
RC-CPHD	8.3444	0.6404
RC-LB	7.2972	1.4842

**Table 9:** Average OSPA distance and running time of different filters versus detection probability when the clutter rate is equal to 6.

Filter Type	Average OSPA Distance (m)	Average Running Time (s)
RC-PHD	16.7275	0.2200
RC-CPHD	6.2314	0.5785
RC-LBM	6.2089	0.7956
